# Construction of an Ortholog Database Using the Semantic Web Technology for Integrative Analysis of Genomic Data

**DOI:** 10.1371/journal.pone.0122802

**Published:** 2015-04-13

**Authors:** Hirokazu Chiba, Hiroyo Nishide, Ikuo Uchiyama

**Affiliations:** 1 Laboratory of Genome Informatics, National Institute for Basic Biology, National Institutes of Natural Sciences, Okazaki, Aichi, Japan; 2 Data Integration and Analysis Facility, National Institute for Basic Biology, National Institutes of Natural Sciences, Okazaki, Aichi, Japan; Kyushu University, JAPAN

## Abstract

Recently, various types of biological data, including genomic sequences, have been rapidly accumulating. To discover biological knowledge from such growing heterogeneous data, a flexible framework for data integration is necessary. Ortholog information is a central resource for interlinking corresponding genes among different organisms, and the Semantic Web provides a key technology for the flexible integration of heterogeneous data. We have constructed an ortholog database using the Semantic Web technology, aiming at the integration of numerous genomic data and various types of biological information. To formalize the structure of the ortholog information in the Semantic Web, we have constructed the Ortholog Ontology (OrthO). While the OrthO is a compact ontology for general use, it is designed to be extended to the description of database-specific concepts. On the basis of OrthO, we described the ortholog information from our Microbial Genome Database for Comparative Analysis (MBGD) in the form of Resource Description Framework (RDF) and made it available through the SPARQL endpoint, which accepts arbitrary queries specified by users. In this framework based on the OrthO, the biological data of different organisms can be integrated using the ortholog information as a hub. Besides, the ortholog information from different data sources can be compared with each other using the OrthO as a shared ontology. Here we show some examples demonstrating that the ortholog information described in RDF can be used to link various biological data such as taxonomy information and Gene Ontology. Thus, the ortholog database using the Semantic Web technology can contribute to biological knowledge discovery through integrative data analysis.

## Introduction

Because of the rapid progress of biotechnology, various types of biological data, including genomic sequences, have been rapidly accumulating; therefore, their effective computational management appears to be a challenging issue in biological data analysis. In particular, the heterogeneity of biological data makes the integration required for data analysis a hard problem. To achieve the integration of such growing heterogeneous data, there is an urgent need for consolidating key information that links biologically related resources to each other.

Among the various biological resources, ortholog information can play a central role in integrating the biological data of multiple species. Originally, orthologs are defined as genes diverged by speciation from an ancestral gene [1], and their biological functions are usually conserved [2]. Thus, ortholog information is a useful resource to link the corresponding genes of different species and transfer the biological knowledge of model organisms to organisms with newly sequenced genomes. In this era where numerous novel genome sequences are being determined, the concept of such computational knowledge transfer is becoming increasingly valuable. In addition, ortholog groups are a vital resource for the comparative analysis of multiple genomes, and they provide a basis for the analysis of phylogenetic profiles (the presence and absence patterns of genes in genomes) [3]. Genomic data integration using ortholog information and comparative analysis based on it are powerful approaches for biological knowledge discovery.

Among the various ortholog databases currently available, our Microbial Genome Database for Comparative Analysis (MBGD) provides a system for users to select specific sets of species to be compared, thus providing a flexible mechanism for finding orthologs [4]. Although MBGD and other ortholog databases provide Web browser interfaces to efficiently retrieve ortholog information and related data, such interfaces are not sufficient for users who want to retrieve various information using the orthology relation as a hub of links.

For the integration of biological data derived from different data sources, the use of the Semantic Web technology [5] is a promising approach [6, 7]. In the Semantic Web, all the information is described in the Resource Description Framework (RDF) [8], in which the Uniform Resource Identifier (URI) assures the uniqueness of each resource worldwide and contributes to valid data integration of data collected from different sources. The Semantic Web technology also provides a search functionality using SPARQL [9] standardized by the World Wide Web Consortium (W3C), which includes a protocol to access the data across the Web. Thus, constructing a database using the Semantic Web that accepts SPARQL queries means that the data are not only locally available but also accessible through arbitrary queries specified by users across the Web. An additional merit of using the Semantic Web is that data modeling is based on ontologies, which define the relations between the terms and work as a translation layer to unite different terminologies used by different resource providers. In the past few years, there has been a continuous effort to apply the Semantic Web to biological databases for enhancing their interoperability [6, 10]. Restructuring the ortholog database as a hub of the biological database network based on the Semantic Web will have a significant impact for biological database integration.

In this study, we have developed an ortholog database using the Semantic Web technology. We proposed a general RDF model for describing ortholog information on the basis of our novel ontology. Using this model, we expressed the ortholog data of MBGD and made them available through the SPARQL endpoint. We showed several examples of SPARQL queries to demonstrate that our ortholog database could work as a hub for integrating several genomic data resources and support knowledge discovery through its search functionalities.

## Results

### General RDF model of ortholog information based on Ortholog Ontology

**Fig 1:**
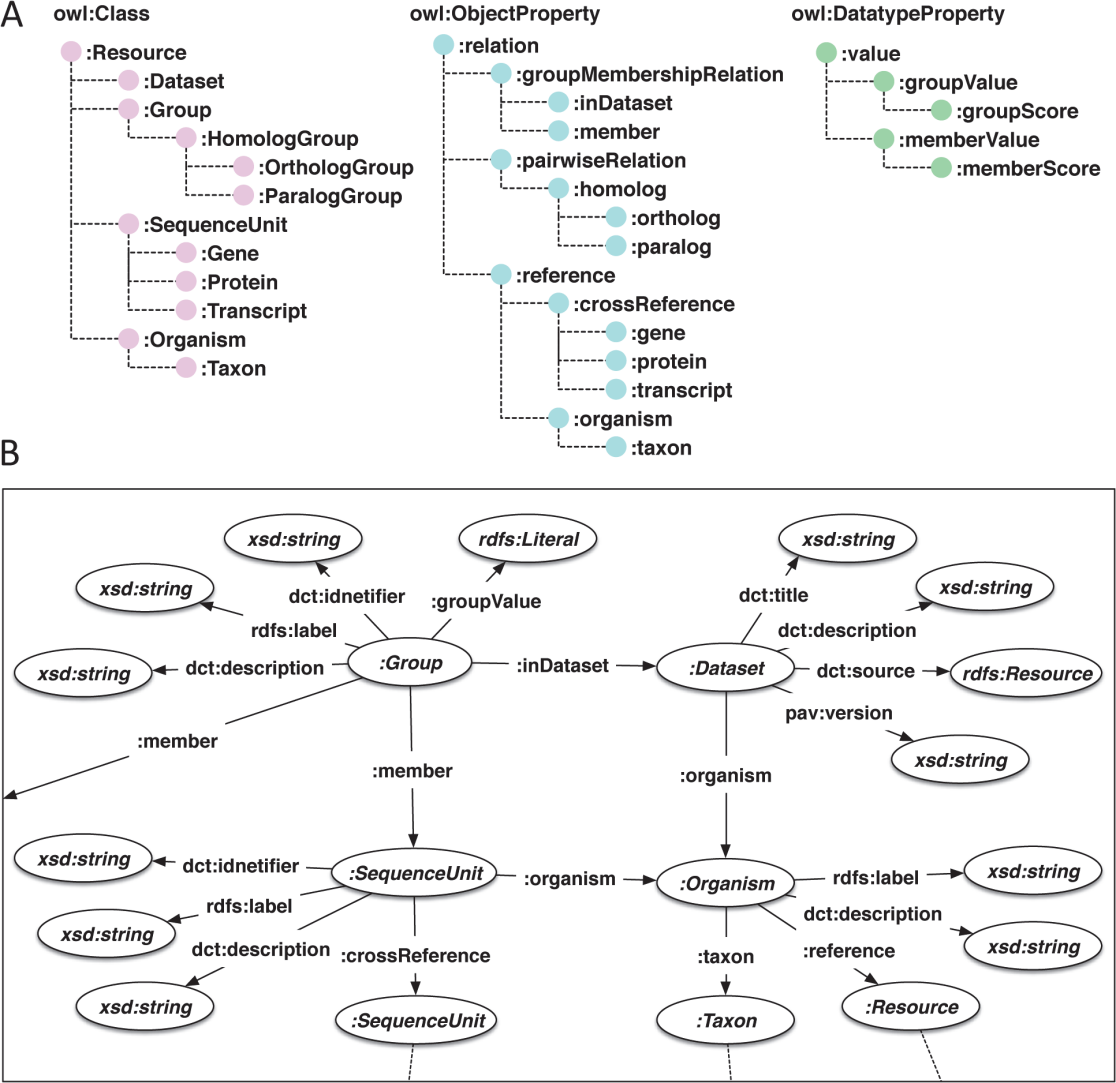
RDF model of ortholog information based on OrthO. (A) Hierarchical structure of classes and properties in OrthO. OrthO includes 12 classes (*owl*:*Class*) and 20 properties (15 of *owl*:*ObjectProperty* and 5 of *owl*:*DatatypeProperty*). (B) Schematic representation of RDF graph structure of ortholog information described using OrthO. The elliptical nodes represent instances of classes. The directed edges represent properties. The dotted lines represent possible links to other resources.

We developed a general RDF model for describing ortholog information. Our model can load different ortholog databases into the same framework for their interoperable use. As the basis of the RDF model, we defined the Ortholog Ontology (OrthO), which comprises the basic terms required for describing the ortholog information (available at http://purl.jp/bio/11/orth). The terms in OrthO are defined using the Web Ontology Language (OWL) [11]: each of the defined terms is either a class to be used for representing a specific group of resources or a property for representing a specific relationship from resources to resources or to data values. The terms in OrthO have hierarchical relationships shown in Fig 1A. On the basis of OrthO, the ortholog information can be described in RDF and thereby is connectable to other resources that are also described in the form of RDF (Fig 1B). The OrthO and other ontologies used in this study are listed in Table 1 with their namespaces and their abbreviated forms (prefix).

**Table 1:** List of ontologies available at the MBGD SPARQL endpoint.

**Ontology title**	**Prefix**	**Namespace**
Ortholog Ontology (OrthO)	orth:	http://purl.jp/bio/11/orth#
An ontology for MBGD	mbgd:	http://purl.jp/bio/11/mbgd#
An ontology for GO annotation	goa:	http://purl.jp/bio/11/goa#
The RDF Concepts Vocabulary (RDF)	rdf:	http://www.w3.org/1999/02/22-rdf-syntax-ns#
The RDF Schema vocabulary (RDFS)	rdfs:	http://www.w3.org/2000/01/rdf-schema#
The OWL 2 Schema vocabulary (OWL 2)	owl:	http://www.w3.org/2002/07/owl#
Dublin Core Metadata Element Set, Version 1.1	dc:	http://purl.org/dc/elements/1.1/
DCMI Metadata Terms	dct:	http://purl.org/dc/terms/
Vocabulary of Interlinked Datasets (VoID)	void:	http://rdfs.org/ns/void#
SKOS Vocabulary	skos:	http://www.w3.org/2004/02/skos/core#
Provenance, Authoring and Versioning (PAV)	pav:	http://purl.org/pav/
Ontological Gene Orthology (OGO)	ogo:	http://miuras.inf.um.es/ontologies/OGO.owl
FALDO: Feature Annotation Location Description Ontology	faldo:	http://biohackathon.org/resource/faldo#
UniProt core ontology	up:	http://purl.uniprot.org/core/
RDF representation of taxonomy	tax:	http://purl.uniprot.org/taxonomy/
RDF representation of GO	go:	http://purl.uniprot.org/go/

This table shows the selected list of ontologies available at MBGD SPARQL endpoint. The prefixes for each ontology used in this study are shown. For the full list and additional details of the available ontologies, see the documentation page of MBGD SPARQL Search (http://mbgd.genome.ad.jp/sparql).

The central class in OrthO is the *OrthologGroup*, which is defined as a set of homologous sequences derived from a common ancestral sequence by speciation. On the other hand, the OrthO also defines *ParalogGroup* as a set of homologous sequences derived from a common ancestral sequence by duplication and *HomologGroup* as a super-class of the *OrthologGroup* and *ParalogGroup*. *SequenceUnit* is a class for generally representing each member of such groups. Typically, *SequenceUnit* is *Gene* or *Protein* in most ortholog databases, but it can be any sequence element between which homology relation can be defined. *SequenceUnit* is linked to its source organism (class *Organism*), which is essential information constituting the ortholog data. To represent the entire set of ortholog groups, the OrthO defines a class *Dataset*. One of the main applications of OrthO is the description of ortholog datasets from different sources within the same framework for integrative use. In such situations, distinct ortholog datasets can be represented as different instances of *Dataset*. Similarly, ortholog classifications for different sets of target organisms or different versions of datasets can also be represented as different instances of *Dataset*.

Among the properties, *member* is the essential property for representing the orthology relation. It typically links *Group* to *SequenceUnit*; however, in the case of the hierarchical grouping of orthologs [12], it may link *Group* to *Group* to form a tree structure, wherein internal nodes are *Group* and leaves are *SequenceUnit*. The property *crossReference* represents the correspondence between two instances of *SequenceUnit* derived from different databases. The property *organism* is used to reference the source organism (*taxon* is used more specifically to reference an NCBI taxonomy ID). The OrthO also includes the properties *ortholog* and *paralog* for describing pairwise relationships between two instances of *SequenceUnit*.

**Fig 2:**
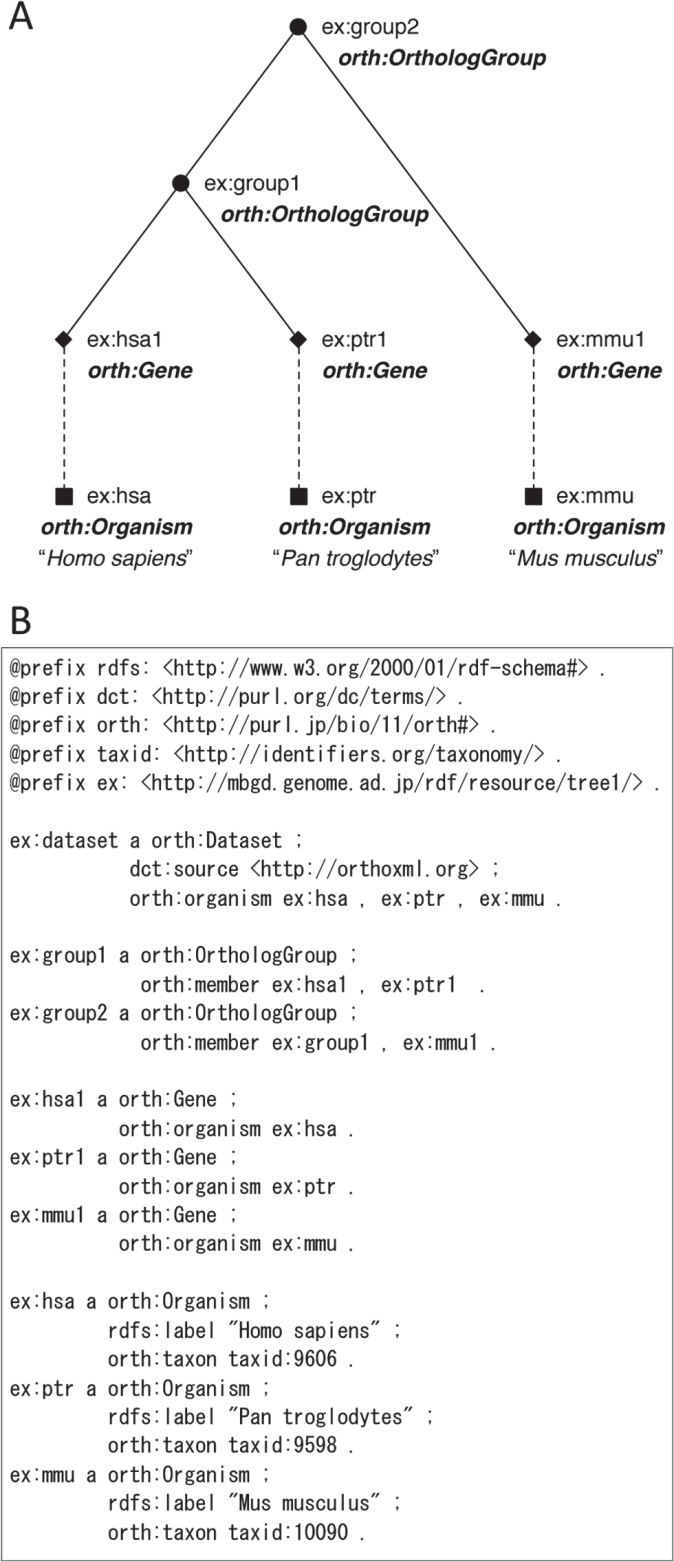
An example orthology relation and its RDF representation. (A) A schematic illustration of an orthology relation from the OrthoXML documentation (http://orthoxml.org/0.3/orthoxml_doc_v0.3.html#trees). Here, each node is assigned a URI and a class that are required for RDF representation. The filled circles representing speciation events are assigned the *orth*:*OrthologGroup* class. (B) RDF representation (Turtle format) of the example shown in A.

An example of ortolog information described by OrthO is shown in Fig 2. Here, a simple orthology relation originally represented in OrthoXML (http://orthoxml.org/0.3/orthoxml_doc_v0.3.html#trees) is illustrated in Fig 2A, and RDF representation of it using OrthO is shown in Fig 2B. This example delineates the essential components in RDF representation using OrthO: the metadata for the dataset (such as the data source), the relation between the group and its members, between genes and organisms, and the references to other resources (in this example, from organism to taxonomy ID). In RDF, each entity is represented by a URI to assure its uniqueness; thus, the namespace for the URIs should be prepared. In this figure, the URIs are expressed in abbreviated forms using prefixes in Turtle format.

### Relation of the OrthO with other models for describing orthologs

The OrthO terms are designed to cover the concepts defined by OrthoXML [13] (http://orthoxml.org), which was proposed as a community standard for formatting ortholog information. The correspondence between the representation in OrthO and that in OrthoXML is summarized in S1 Table. Because of their correspondence, ortholog information described in OrthoXML can also be described using OrthO (see Fig 2 for a simple example). The description of a more complex example (http://orthoxml.org/0.3/orthoxml_doc_v0.3.html#example) using OrthO is shown in S1 Fig (S1 Dataset includes the RDF file).

In comparison to a preceding study of ontology for orthology (OGO) [14], our model is designed to be more general and conforming to various ortholog databases. In particular, in our OrthO, sequence units constituting orthologs may be a gene, a protein, a transcript, or even a newly defined element on genome sequences (such as orthologous domains [12, 15, 16] or regulatory regions). Further, OrthO can be applied to a hierarchical grouping of orthologs and paralogs created by assigning a speciation or duplication event to each node [17] (a simple example is shown in Fig 2), enabling a more elaborate description of evolutionary relationships between group members. Correspondence between the representation in OrthO and that in OGO is shown in S1 Table. Some representations, including the metadata for the dataset and the scores of grouping, are not considered in OGO. To fully exploit the existing ortholog dataset described in OrthoXML, these representations are necessary.

In addition to its unique characteristics, the OrthO has compatibility and interchangeability to other ontologies, which increases its usability. To achieve the compatibility for the concepts commonly existing in the OrthO and OGO, the corresponding classes or properties are associated in the OrthO, thus enabling automatic translation between terminologies when inference is enabled in RDF stores. For example, *orth*:*member* and *ogo*:*hasOrthologous* are associated by *owl*:*equivalentProperty*; thus, ortholog information described in the OrthO can be searched by OGO. Furthermore, the terms in OrthO are also associated with other generally used ontologies such as Sequence Ontology (SO) [18] and Semanticscience Integrated Ontology (SIO) [19].

### Ortholog database implementation using RDF

Whereas the OrthO can describe basic information commonly contained in most ortholog datasets, each dataset often contains database-specific concepts. The OrthO can be extended to describe such database-specific concepts. For describing MBGD data, we have constructed a database-specific ontology for MBGD (MBGD-O, available at http://purl.jp/bio/11/mbgd, see S2 Fig for hierarchical structure). The ontology includes specific terms such as *mbgd*:*Domain* to represent a sub-sequence of a protein as a sequence unit of classification and *mbgd*:*Chromosome* to represent a chromosome containing each gene. Such terms are designed to conform to the existing architecture of the MBGD database, enabling easy conversion from the existing database files to their RDF representation. The graph structure of RDF described by OrthO and MBGD-O is shown in S3 Fig In the MBGD-O, terms related to orthologs are defined based on the OrthO (i.e., associated with the OrthO terms using *rdfs*:*subClassOf* or *rdfs*:*subPropertyOf*). Because of these associations between the different levels of ontologies, the database can be searched either by database-specific terms (e.g., *mbgd*:*uniprot* representing a cross-reference to UniProt) or more general terms (e.g., *orth*:*crossReference*). Because the OrthO can provide a basis shared by different ontologies, the ortholog data described in different terminologies can be compared/merged by way of the OrthO.

To further demonstrate the ability of our model to describe the ortholog information, we applied our model to other ortholog databases. Among the previously developed ortholog databases [20, 21], many databases have been represented using OrthoXML. Such data can be described by our model. On the other hand, the eggNOG database [22] contains positional information of orthologous regions at sub-gene level. This does not conform to OrthoXML but is common to MBGD. In fact, we could describe the data obtained from the eggNOG database using the OrthO and MBGD-O to create the RDF version of eggNOG, which was used for comparison with our database below (see the section comparing ortholog information from different data sources).

To realize the retrieval of ortholog information described in RDF, we used an RDF store, Virtuoso [23]. The list of stored graphs and number of triples contained in each of them are shown in Table 2. The total number of triples stored is 1,150,394,708. The RDF data stored in Virtuoso can be retrieved by SPARQL across the Web using HTTP (http://sparql.nibb.ac.jp/sparql).

**Table 2:** List of the datasets available at the MBGD SPARQL endpoint.

**Dataset title**	**Graph name**	**Triples**
MBGD ortholog groups	http://mbgd.genome.ad.jp/rdf/resource/2014-01_default	76,155,196
MBGD genes	http://mbgd.genome.ad.jp/rdf/resource/2014-01_gene	686,902,009
MBGD organisms	http://mbgd.genome.ad.jp/rdf/resource/2014-01_organism	31,397
MBGD chromosomes and plasmids	http://mbgd.genome.ad.jp/rdf/resource/2014-01_nucseq	6,796,757
Cross-references from MBGD to UniProt	http://mbgd.genome.ad.jp/rdf/resource/2014-01_xref_uniprot	8,012,666
eggNOG COG	http://mbgd.genome.ad.jp/rdf/resource/eggnog_3.0_COG	42,787,220
eggNOG NOG	http://mbgd.genome.ad.jp/rdf/resource/eggnog_3.0_NOG	21,469,150
eggNOG proteins	http://mbgd.genome.ad.jp/rdf/resource/eggnog_3.0_protein	24,572,358
eggNOG organisms	http://mbgd.genome.ad.jp/rdf/resource/eggnog_3.0_organism	1,144
UniProt-GOA	http://mbgd.genome.ad.jp/rdf/resource/uniprot-goa	274,338,183

For detailed information on the datasets, including their derivation, see the documentation page of MBGD SPARQL Search (http://mbgd.genome.ad.jp/sparql).

**Fig 3:**
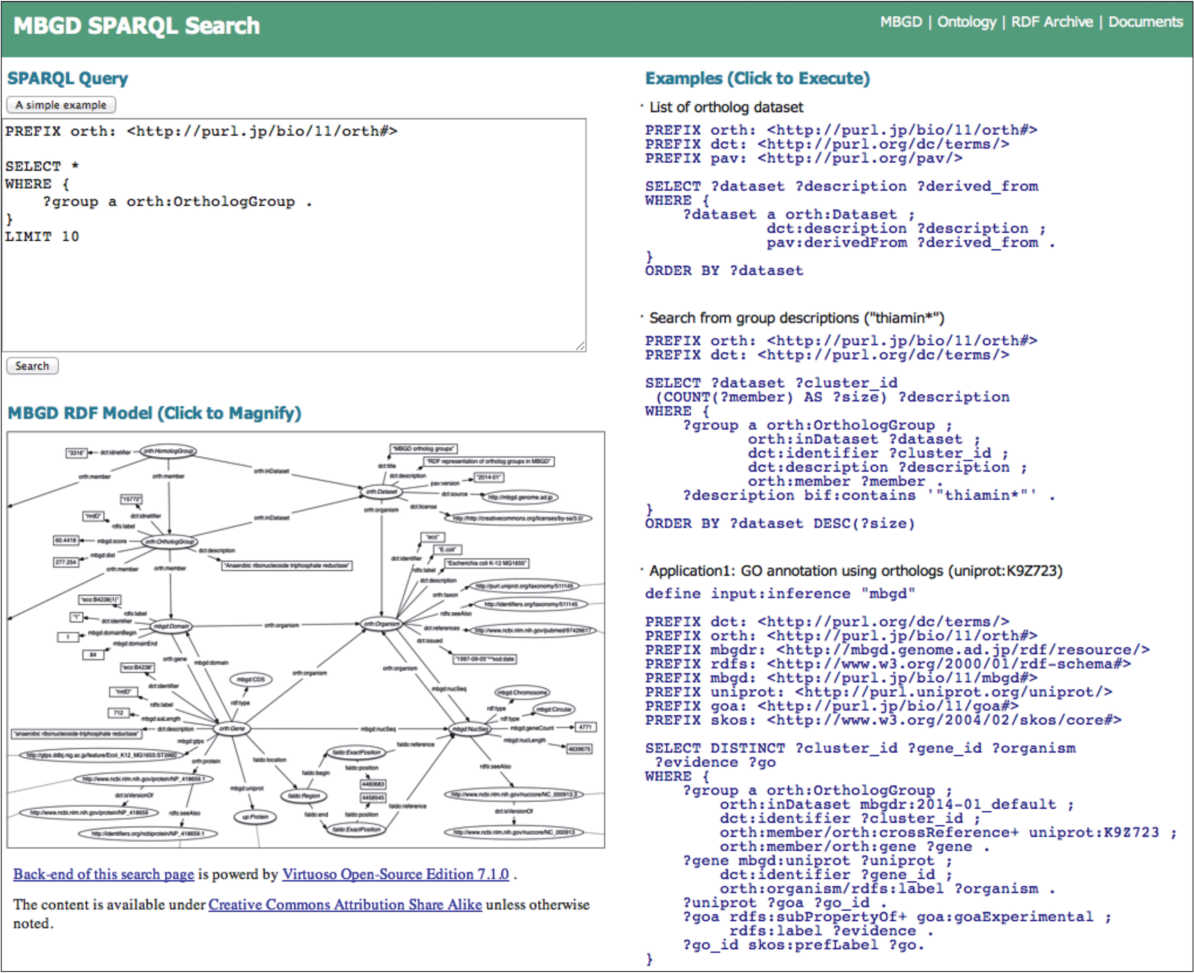
The portal page of MBGD SPARQL Search.

To provide users with easy access to the RDF data, we created a portal site for searching the database (http://mbgd.genome.ad.jp/sparql), which includes a schematic illustration of the RDF structure, query examples, ontology downloads, RDF archives, and documentations (Fig 3). On the portal site, the retrieval of ortholog information can be executed by entering a SPARQL query in the text box, and typical example queries are shown alongside. Those examples are clickable and provide an easy test environment for starters. All examples in this paper are available at this portal site. Experienced users can access the SPARQL endpoint from their own programs via HTTP access. Alternatively, users can specify the SPARQL endpoint as the target of *SERVICE* keyword in a federated SPARQL query. In addition, access to each resource URI under the namespace of MBGD is dynamically converted into a query to the SPARQL endpoint (see Methods), enabling an instant browse of MBGD RDF. The MBGD RDF data created in this work is downloadable from http://mbgd.genome.ad.jp/rdf/archive/ and available under Creative Commons Attribution Share Alike (CC BY-SA 3.0).

### Retrieving ortholog information of a specific protein

A typical use of an ortholog database is transferring functional annotations from known genes in model organisms to genes of unknown function in other organisms, on the basis of the conjecture that orthologs are usually functionally conserved. To demonstrate such an application in our database, we showed a query to retrieve ortholog information of a specified protein. Here, we specified a UniProt ID to obtain ortholog information. For describing functional categories of genes, we used Gene Ontology (GO) [24]. The UniProt GO Annotation (UniProt-GOA) database [25] (http://www.ebi.ac.uk/GOA) provides GO term assignment to proteins with evidence codes (http://www.geneontology.org/GO.evidence.shtml). We created an ontology for GO annotation (GOA-O, Table 1, http://purl.jp/bio/11/goa) and described UniProt-GOA data in RDF using it (Table 2). If some model organisms have experimentally verified GO annotations, we can transfer such a validated annotation to orthologs of other organisms.

**Fig 4:**
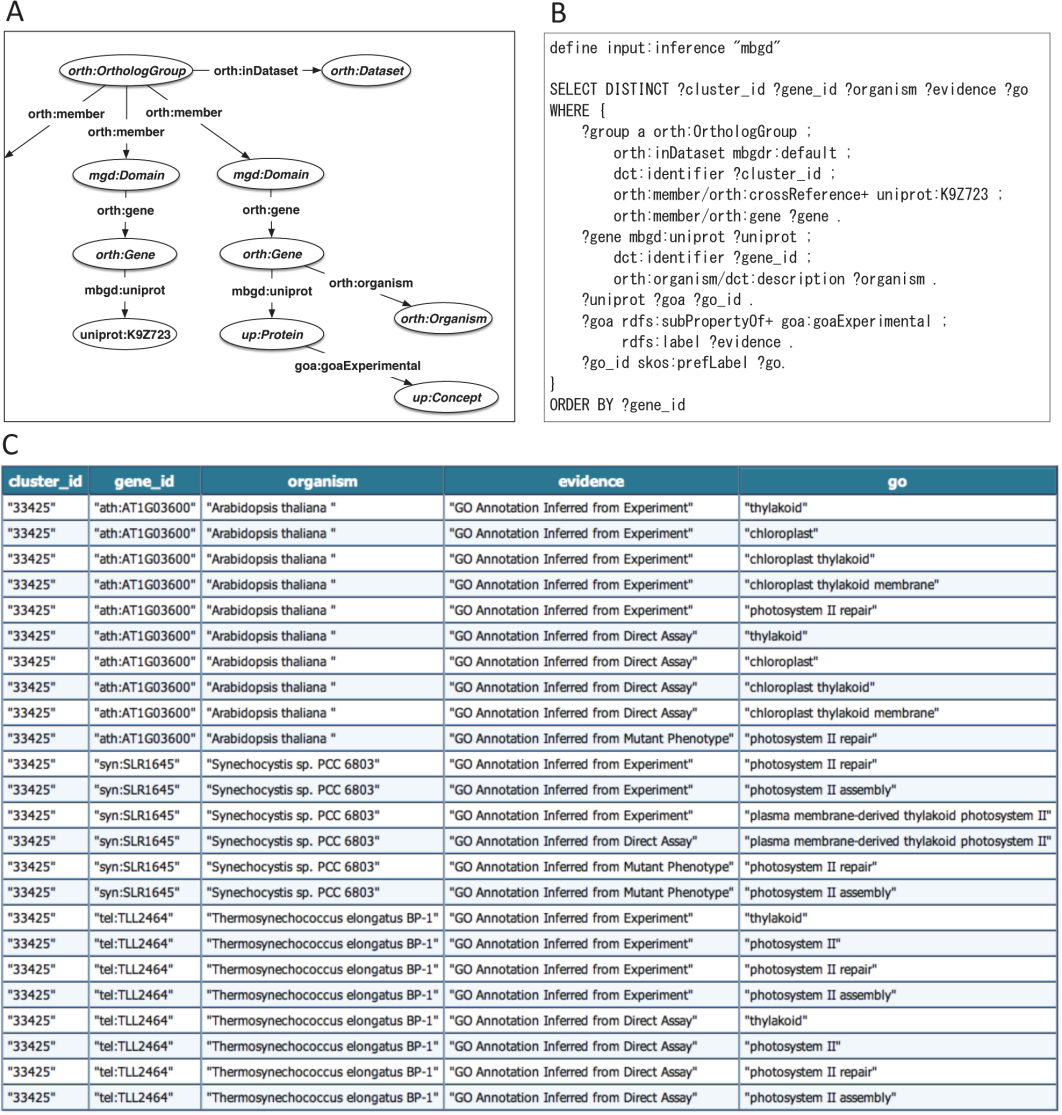
Retrieval of ortholog information of a specific protein. (A) Schematic diagram of the RDF graph structure related to the query in B. The elliptical nodes represent resources. Specifically, the shaded elliptical nodes where classes are shown in italics represent the instances of the classes. In the unshaded elliptical node, the URI of the resource is directly shown. (B) SPARQL query to get GO annotation of an ortholog group. The prefix declarations are omitted for readability; the full description of the SPARQL query is included in S1 Dataset. (C) Search results of the query shown in B.

Fig 4 shows an example SPARQL query to retrieve experimentally verified GO annotations assigned to some orthologs of the query protein UniProt K9Z723; Fig 4A shows the RDF data structure related to this query and Fig 4B shows the SPARQL code. While this protein of *Cyanobacterium aponinum* PCC 10605 does not have any GO annotation with experimental evidence codes, the ortholog information provides corresponding proteins of other organisms, including *Synechocystis* sp. PCC 6803, which have GO annotations, including “photosystem II repair,” with experimental evidence codes such as “GO Annotation Inferred from Experiment” and “GO Annotation Inferred from Mutant Phenotype,” which are represented by sub-properties of *goa*:*goaExperimental* (Fig 4C).

### Comparing ortholog information from different data sources

**Fig 5:**
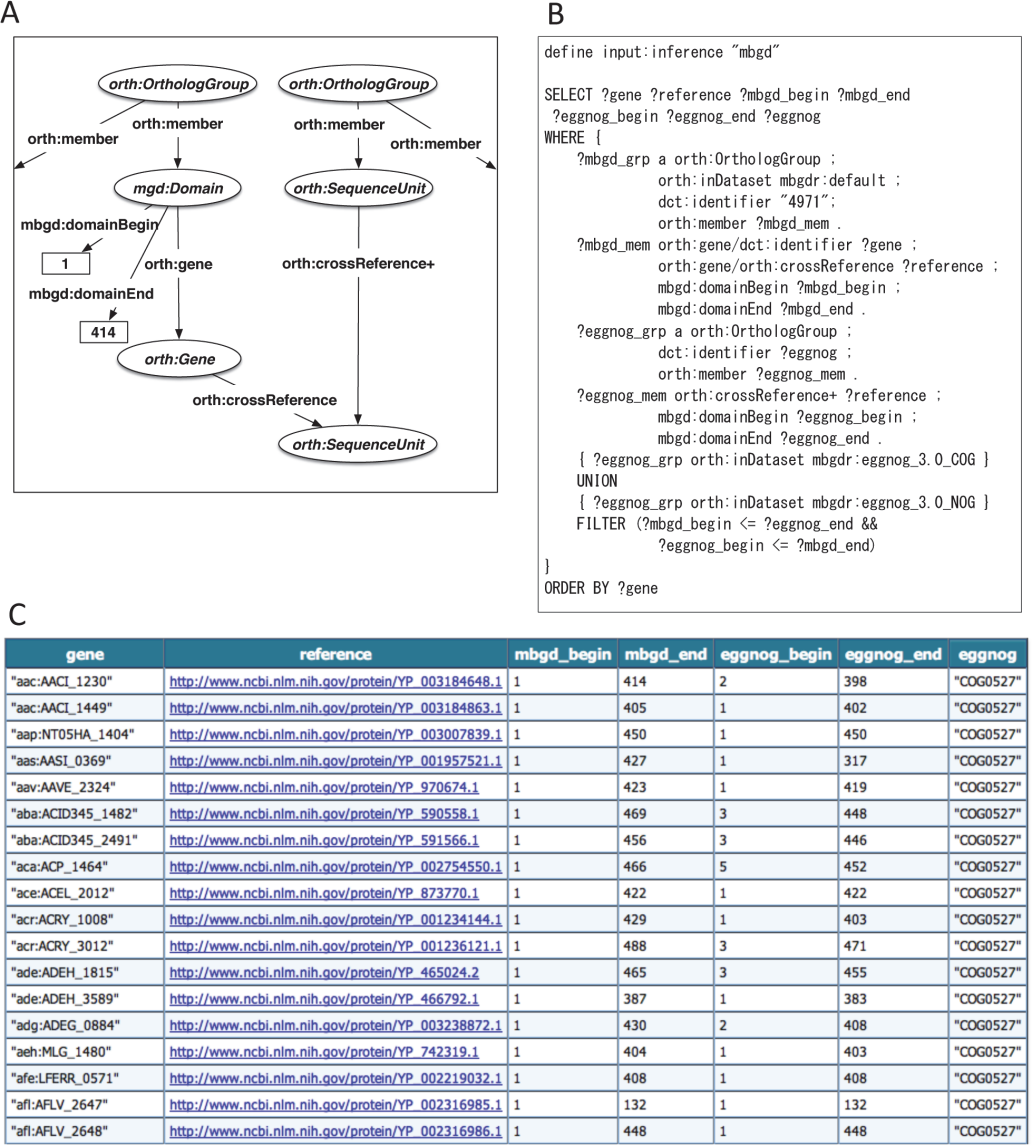
Comparison of ortholog information from different data sources. (A) Schematic diagram of the RDF graph structure related to the query in B. The elliptical nodes represent instances of classes. The rectangular nodes represent literals (integers in this example). (B) SPARQL query to compare orthologs between MBGD and eggNOG. The first line enables the inference based on sub-class and sub-property relations (see Methods). (C) Search results of the query shown in B.

There are various ortholog databases that are constructed based on different methods and different sets of genomes. The users’ concerns when using them may include comparing or merging ortholog groups derived from different data sources. However, the differences in the resource IDs (e.g., gene IDs and organism IDs) between them could hamper this task because finding identical members between corresponding groups is not straightforward. In our framework, even if independent gene IDs are used in different ortholog databases, cross-reference information assigned to each gene in each database can indirectly create a linkage between the corresponding genes through a common cross-reference. To test the comparability between data from different sources, we used the RDF version of ortholog information of eggNOG in addition to MBGD. The SPARQL query shown in Fig 5 finds eggNOG clusters corresponding to a given MBGD cluster. Although gene IDs in MBGD and those of corresponding genes in eggNOG are different, cross-references to the common database entry IDs, in this case UniProt IDs or RefSeq IDs, make it possible to interlink corresponding entries in MBGD and eggNOG. Although the query in Fig 5B does not explicitly specify any database name for cross-reference, it can find corresponding entries through either UniProt ID or RefSeq ID because the general property *orth*:*crossReference* is a super-property of both *mbgd*:*uniprot* (referring to UniProt ID) and *mbgd*:*protein* (referring to RefSeq ID) and *orth*:*crossReference+* allows arbitrary times (one or more) of any cross-references. Thus, the abstraction mechanism based on the ontology enhances the integration of different datasets by hiding implementation details in each database. The query compares the MBGD and eggNOG cluster members (both of which are domains, i.e., sub-sequences of proteins), and finds overlapping segments within the same genes (Fig 5C). It is possible to make more useful linkages between ortholog groups from different databases using a more complicated query (see “example2a.rq” in S1 Dataset); the number of common members is divided by each group size to produce overlap ratios, which are then used to define the relations between the ortholog groups, such as equivalent, subgroup, and supergroup with a similar criterion to that for the cross-reference section in the MBGD database [26].

### Correlating gene functions with phylogenetic pattern using ortholog information

**Fig 6:**
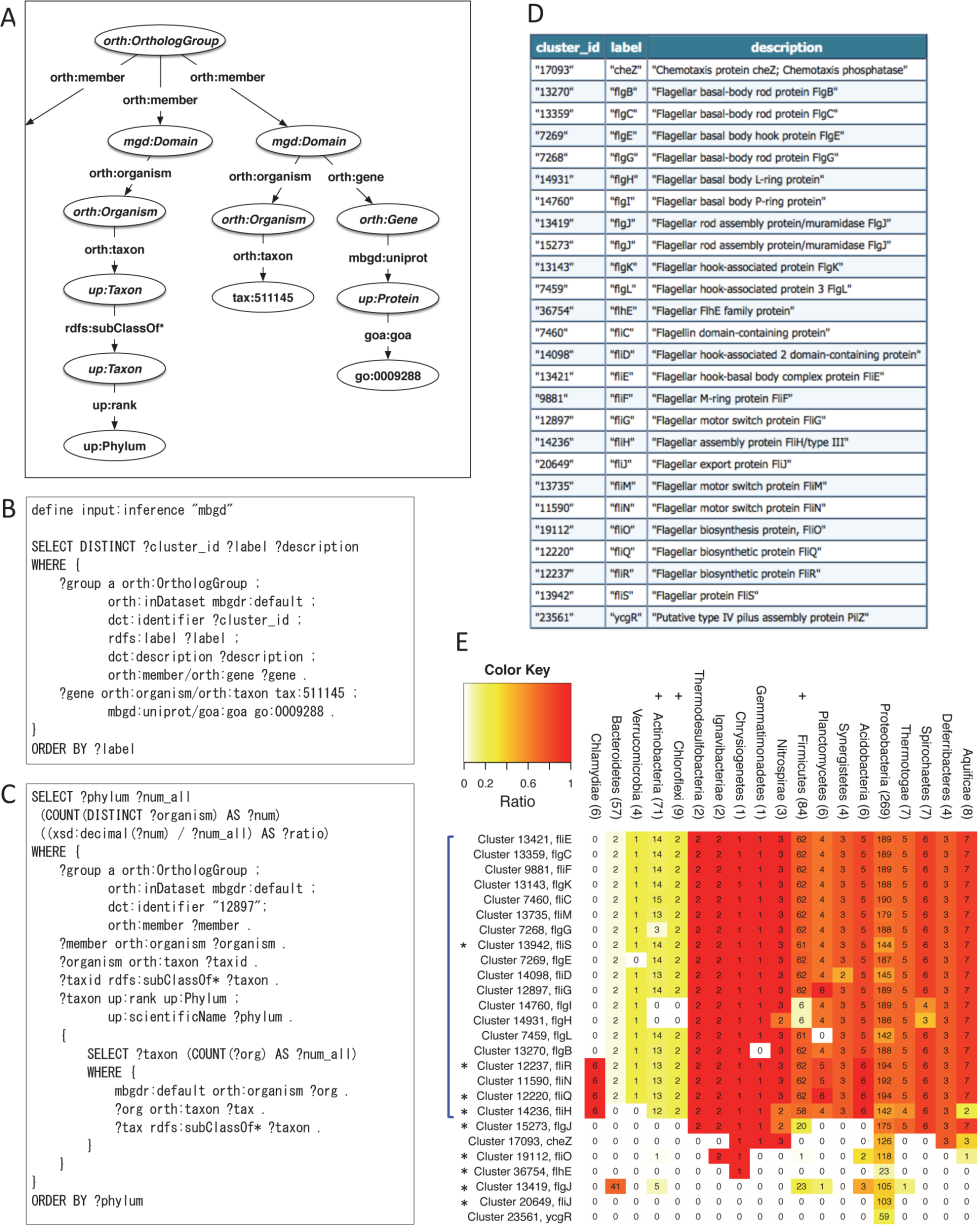
Retrieval of phylogenetic patterns of orthologs related to a specific function. (A) Schematic diagram of the RDF graph structure related to the queries in B and C. (B) SPARQL query to get MBGD clusters including members related to the GO term GO:0009288 (bacterial-type flagellum). (C) SPARQL query to obtain organisms that contain members of an ortholog group. (D) Search results of the query shown in B. (E) The results obtained from the queries shown in B and C visualized using R (the R source code is included in S1 Dataset). The number of target organisms in each phylum is shown in parenthesis. After obtaining the output from R, the phyla containing gram-positive bacteria (+) and genes functioning in the flagellar export system (*) are marked, and the blue line was added to represent clusters with relatively wide organismal distribution (in at least 16 phyla).

As the third application, we showed queries that find relationships between gene functions and taxonomy of organisms by tracing the linkages in RDF (Fig 6). As an ortholog group is connected to a set of organisms as well as a set of functional categories through its members in RDF (Fig 6A), it can link between a gene function and a set of organisms having that function. If a functional category (GO term) is specified, we can obtain genes assigned that functional category and then the ortholog groups containing them. For example, the query shown in Fig 6B searches for MBGD clusters that include members assigned a specific GO term, GO:0009288 (bacterial-type flagellum) in *Escherichia coli* K-12 MG1655 (NCBI taxonomy ID 511145). The obtained list of MBGD clusters is shown in Fig 6D. One of the clusters (in this example, cluster 12897, namely fliG) is specified in the query shown in Fig 6C, which searches for organisms included in this cluster and returns the phylogenetic pattern summarized at a given taxonomic rank (in this example, phylum) using the hierarchical taxonomic classification.

Using the R environment (see Methods), the SPARQL queries in Fig 6B and 6C were sequentially executed to obtain the phylogenetic pattern of the clusters, and then the results were visualized as a heat map (Fig 6E). Out of the 26 clusters, 19 showed relatively wide organismal distribution ranging in at least 16 phyla of bacteria, whereas the other 7 clusters distributed in smaller ranges, including at least *Proteobacteria* that *E*. *coli* belongs to. Among the former 19 clusters with overall similarity in distributions, slight differences were observed. The differences in the phylogenetic pattern could reflect species- or taxon-specific functions of the bacterial flagellum genes, although they basically have known functions of bacterial motility. Specifically, clusters 14760 (flgI) and 14931 (flgH) tend to be missing in the phyla including gram-positive bacteria (marked by + in Fig 6E). Here, flgI products constitute the P (peptidoglycan) ring in the peptidoglycan layer and flgH products constitute the L (lipopolysaccharide) ring in the outer membrane. The molecular characteristics of these gene products correlate with the phylogenetic pattern that these genes are missing in the phyla including gram-positive bacteria that have a thick peptidoglycan layer and lack an outer membrane [27]. Notably, 4 clusters (fliR, fliN, fliQ and fliH) contain genes from *Chlamydiae*, whereas other clusters do not. Considering that *Chlamydiae* are non-motile bacteria, this result suggests that these genes could be related to functions other than motility. In fact, these genes are known components of the type III secretion system, which delivers effectors into eukaryotic cells and is evolutionarily related to the bacterial flagellum [28, 29].

### Performance of loading and querying to the RDF store

The performances of the existing programs implementing the Semantic Web technology have recently been improved. These improvements include updates of Virtuoso. However, the performances need to be further improved. With respect to hardware performance, high-speed drives such as solid-state drives can enhance the database performance. We measured the time required for loading and querying to the Virtuoso installed on a solid-state drive. As a result, the loading of the dataset “MBGD chromosomes and plasmids” in Table 2 (6,796,757 triples) took 28.4 s, indicating that the loading speed was 14.4 million triples per minute. The execution time required for the queries is shown in Table 3.

**Table 3:** Time required for executing SPARQL queries.

**Query**	**Time (s)**
Fig 4B	1.2
Fig 5B	3.3
Fig 6B	1.6
Fig 6C	19.1

## Discussion

In this study, we developed an RDF model for integrating the genomic data of multiple organisms using the ortholog information as a hub. The RDF model is constructed based on the OrthO, which is a compact ontology for general use but is also designed to be extensible to cover database-specific concepts. We demonstrated the usefulness of our model in the integrative analysis of multiple genomes by describing our MBGD database in RDF and linking the data to various other resources. Our database can work as part of the worldwide database network through an application designed using Semantic Web technologies, including RDF, SPARQL, and OWL. Although our current raw-level SPARQL query interface is mainly designed for those familiar with database technologies, including database providers, application developers, and bioinformatics experts, an application using Semantic Web technologies with a more user-friendly interface can facilitate the use of our database by a broader range of biomedical researchers as part of the worldwide database network.

With respect to database integration, this study addressed several aspects. One aspect is to create linkages between various biological resources on the basis of the connective nature of RDF. Another is to use the characteristics of orthologs as a hub of links between organisms. The third is to utilize ontologies that have the ability to unite different terminologies.

One of the main advantages of using the Semantic Web technology instead of conventional technologies is that databases get highly connectable to other resources, both locally and globally on the Web. More specifically, improved local connectivity means that when several graphs are imported into an RDF store, a merged graph is automatically generated, and the concatenation of the RDF files immediately produces a valid merged RDF file (in the case of specific formats such as Turtle [30] and N-triples [31]). Moreover, improved global connectivity means that the RDF data are accessible through SPARQL across the Web, which will ultimately transform the Web into a big database cooperatively constructed by developers worldwide. The Semantic Web includes another form of global access that does not require the SPARQL endpoint; direct access to a resource URI can be used as an easy way of connecting the resource information if it returns data. Our database not only accepts access to the SPARQL endpoint but also provides an easy access without SPARQL by specifying URIs on the web browsers (see Methods).

Database integration consequently enables the comparison of corresponding data from different data sources that otherwise could not be easily compared. In this study, we compared the ortholog groups in different classification systems (i.e., MBGD and eggNOG) by comparing the members in each group. Finding the corresponding groups (or subgroups) through such a comparison of their members is not limited to the case of ortholog group comparison but instead is a general issue for other types of groupings such as functional categories. In general, comparing members between different grouping systems produces new relations between the groups (or between the concepts behind each group, such as GO terms). Even if different grouping systems use specific types of resource IDs, we can identify the corresponding genes in RDF as those having links to a common reference through *orth*:*crossReference* or its sub-properties.

Ortholog information, by nature, has a hub structure that connects corresponding genes between organisms. Besides, the evolutionary relationships between orthologs or organisms are often represented in tree structures. Thus, the representation of ortholog information and their evolutionary relationship using graphs is a straightforward approach. In fact, we demonstrated that the RDF version of ortholog and taxonomy information is useful for analyzing the genomic contents of multiple organisms (Fig 6). The use of recursive property paths of SPARQL 1.1 functionalities (such as *rdfs*:subClassOf* and *orth*:*member+*) is suitable for the retrieval of data by traversing a hierarchical structure and enhances the usefulness of the RDF version of the ortholog database.

Because of the existence of various formats for ortholog information, there is a need for standards in the orthology field. OrthoXML was developed as the first standard format of orthology information. In comparison to XML, the RDF representation has several merits. It enables data merging by just concatenating the collected files and is searchable by complex queries. Besides, RDF has a high flexibility and extensibility. For example, some ortholog databases, such as MBGD and eggNOG, include positional information within genes for which sequence homology is detected. OrthoXML is not suitable for expressing such a concept of domain-level orthology. Although OrthO basically conforms to OrthoXML, it can be extended to represent database-specific concepts, such as domain-level orthology. Thus, RDF representation using OrthO is a good candidate for a more general model for describing ortholog information.

Although only a universal terminology is ideal in terms of worldwide database integration, each research group may propose their own terminology depending on their specific scopes, resulting in the existence of equivalent concepts in different terminologies. However, the problem of different terminologies could be solved by translating them. Here, we created an association between OrthO, OGO, SO, and SIO. Besides, each database has its specific concepts and it will be convenient for the maintainer to treat the database by designing the terminologies that reflect the concepts. In this study, we defined the MBGD-specific terms under the general terms of OrthO. Thus, the general terminology worked as an abstraction layer over the database-specific terms. Searching the database using the general terminology as abstraction enables the search against similar data in different databases, thus enabling crossover search and integrative analysis. The Semantic Web, with this abstraction mechanism, reduces the burden of data integration even though individual database developers implement their databases differently according to their own philosophies, which is quite common in cutting-edge scientific fields.

## Conclusions

We developed a general RDF model for describing ortholog information on the basis of an ontology OrthO. The model enables the integration of functional information for multiple organisms. Besides, the ortholog information from different data sources can be compared using the OrthO as a shared ontology. By representing the data in this RDF model, the ortholog database can work as a hub structure for biological databases in the Semantic Web, and it will contribute to knowledge discovery through integrative data analysis.

## Methods

### Creation of ontologies

OrthO, MBGD-O, and GOA-O were created on the ontology editor, Protégé [32] Desktop 4.3 OS X application bundle, which was obtained from http://protege.stanford.edu. The ontology files were saved in Turtle format. Afterwards, the ontology files were manually edited using text editors. The created ontologies are available at http://mbgd.genome.ad.jp/ontology/. For covering the concepts defined by OrthoXML, we inspected an example described in the OrthoXML documentation (http://orthoxml.org/0.3/orthoxml_doc_v0.3.html) and listed the representations used therein (S1 Table). The terms to be included in OrthO were then determined according to this list. Among these terms, we designed a hierarchical class structure, if necessary.

### Preparation of datasets in RDF

The ortholog information and the related data about genes, genomes, and organisms included in MBGD release 2014–01 [4] were converted to RDF (downloadable at http://mbgd.genome.ad.jp/rdf/archive in Turtle format) and loaded to Virtuoso. Creation of the RDF version is not a replacement of the previously developed MBGD system but an extension of it. When a new MBGD dataset is released, the RDF version of the MBGD dataset will be updated. Currently, we provide only one MBGD ortholog dataset (“default dataset”) with the URI http://mbgd.genome.ad.jp/rdf/resource/default, which corresponds to the default ortholog table in MBGD. When multiple datasets are introduced in our database in the future, separate URIs will be set up to distinguish the datasets. To distinguish the target dataset, a property *orth*:*inDataset* can be used. In this study, COG and NOG clusters included in the eggNOG database v3.0 [33] were concatenated and designated as eggNOG. Taxonomy information and gene ontology represented in RDF were downloaded from the UniProt [34] FTP site (ftp://ftp.uniprot.org/pub/databases/uniprot/current_release/rdf/taxonomy.rdf.gz, ftp://ftp.uniprot.org/pub/databases/uniprot/current_release/rdf/go.rdf.gz), and loaded to Virtuoso. GO annotation with evidence codes was downloaded from the UniProt-GOA database (ftp://ftp.ebi.ac.uk/pub/databases/GO/goa/UNIPROT/gene_association.goa_uniprot.gz). The converters from the original data to RDF were written in Perl. Raptor RDF Syntax Library (http://librdf.org/raptor/) version 2.0.13 was used to count the numbers of triples of RDF files.

### Settings of the RDF store

RDF data and ontologies were loaded to an RDF store and made accessible through the SPARQL endpoint. Virtuoso Open-Source Edition (http://www.openlinksw.com/dataspace/doc/dav/wiki/Main/) 7.1.0 was installed into Linux and was used as an RDF store and the SPARQL endpoint. *DB*.*DBA*.*TTLP_MT()* function was used to load the data in Turtle format and *DB*.*DBA*.*RDF_LOAD_RDFXML_MT()* function for RDF/XML format, through the *isql* interface of Virtuoso. To load RDF in Turtle format containing triples larger than 10 million triples, the triples were divided into smaller files containing less than 10 million triples and loaded in parallel using the *ld_dir()* and *rdf_loader_run()* functions with a parallelization degree of four. To make inference rules defined in ontologies executable in Virtuoso, the *rdfs_rule_set()* function was used through the *isql* interface of Virtuoso. To enable inference for a SPARQL query to Virtuoso, the following line should be specified at the beginning of the query,

define input:inference “mbgd”

where “mbgd” is the rule set including OrthO, MBGD-O, and GOA-O. There is another rule set “ontologies” that includes all the ontologies stored in the MBGD SPARQL endpoint. The functionality of Virtuoso was restricted by setting several options. The maximum number of returned results was set to 10,000. The maximum system memory usage was set to 8 GB. Virtuoso is accessible as the MBGD SPARQL endpoint at http://sparql.nibb.ac.jp/sparql.

### Testing the SPARQL endpoint

When calculating the query execution time, Virtuoso was restarted before each calculation to refresh the cache. To analyze the data by accessing the SPARQL endpoint from local computers, the SPARQL package (http://cran.r-project.org/web/packages/SPARQL/) of R (http://www.r-project.org/) was used as the SPARQL client.

### Instant browse of MBGD RDF

Each resource URI under the namespace of http://mbgd.genome.ad.jp/rdf/resource/ was made dereferenceable by converting the HTTP access into a query to the SPARQL endpoint using a Perl CGI. For example, HTTP access to http://mbgd.genome.ad.jp/rdf/resource/gene/eco:B0002 is converted to the following SPARQL query,

SELECT? subject? predicate? object

WHERE {

{<http://mbgd.genome.ad.jp/rdf/resource/gene/eco:B0002>? predicate? object}

UNION

{? subject? predicate <http://mbgd.genome.ad.jp/rdf/resource/gene/eco:B0002>}

}

which enables the instant browse of the RDF graph under the MBGD RDF name space by clicking the URI on Web browsers.

## Supporting Information

S1 Fig RDF representation of the OrthoXML example. This orthology relation from OrthoXML documentation (http://orthoxml.org/0.3/orthoxml_doc_v0.3.html#example) includes more complex information than the simple example shown in Fig 2. It includes more metadata and references. Further, it includes scores for the groups and group members. To incorporate a score for a group, the score value is directly assigned to *Group* through a sub-property of *groupScore*. On the other hand, assigning a score directly to *Gene* is not appropriate, because *Gene* resource is shared by different clustering results while the score depends on clustering. Instead, *Group* is linked to a node representing a specific group member with a reference to *Gene*, and the node is assigned the score through a sub-property of *memberScore*.
(PDF)

S2 Fig Hierarchical structure of classes and properties in MBGD-O. MBGD-O includes 16 classes (*owl*:*Class*) and 25 properties (4 of *owl*:*ObjectProperty* and 21 of *owl*:*DatatypeProperty*). Terms of OrthO are shown in gray.
(PDF)

S3 Fig Schematic diagram of the RDF representation of MBGD data. The elliptical nodes represent resources. Specifically, the shaded elliptical nodes where classes are shown in italics represent instances of the classes. In the unshaded elliptical nodes, the URIs of the resources are directly shown. The rectangular nodes represent literals. The directed edges represent properties. The dotted lines represent possible links to other resources.
(PDF)

S1 Table Correspondence between the representation in OrthO and those in other models. (PDF)

S1 Dataset A compressed file, including an RDF file, SPARQL queries, and an R source code. The compressed file includes the RDF representation of the OrthoXML example, SPARQL queries, the R source code for executing the SPARQL queries, and a README file.
(TGZ)
