# Supplementary material for: Construction of an Ortholog Database Using the Semantic Web Technology for Integrative Analysis of Genomic Data
Source: PLoS One. 2015 Apr 13;10(4):e0122802. doi: 10.1371/journal.pone.0122802 (PMC4395280; doi:10.1371/journal.pone.0122802)
Supplement: S1 Fig — This orthology relation from OrthoXML documentation (http://orthoxml.org/0.3/orthoxml_doc_v0.3.html#example) includes more complex information than the simple example shown in Fig 2. It includes more metadata and references. Further, it includes scores for the groups and group members. To incorporate a score for a group, the score value is directly assigned to Group through a sub-property of groupScore. On the other hand, assigning a score directly to Gene is not appropriate, because Gene resource is shared by different clustering results while the score depends on clustering. Instead, Group is linked to a node representing a specific group member with a reference to Gene, and the node is assigned the score through a sub-property of memberScore. (PDF) [file pone.0122802.s001.pdf]

```
@prefix rdfs: <http://www.w3.org/2000/01/rdf-schema#> .
@prefix xsd: <http://www.w3.org/2001/XMLSchema#> .
@prefix dct: <http://purl.org/dc/terms/> .
@prefix void: <http://rdfs.org/ns/void#> .
@prefix pav: <http://purl.org/pav/> .
@prefix orth: <http://purl.jp/bio/11/orth#> .
@prefix : <http://mbgd.genome.ad.jp/rdf/resource/orthoxml_example/> .
@prefix taxid: <http://identifiers.org/taxonomy/> .
@prefix wgene: <http://www.wormbase.org/db/gene/gene?name=> .
@prefix wprot: <http://www.wormbase.org/db/seq/protein?name=WP:> .
@prefix hgene: <http://Dec2008.archive.ensembl.org/Homo_sapiens/geneview?gene=> .
@prefix hprot: <http://Dec2008.archive.ensembl.org/Homo_sapiens/protview?peptide=> .

:dataset
    a orth:Dataset ;
    dct:title "Examle of OrthoXML file" ;
    dct:description "Stripped down version of a real InParanoid 7.0 file." ;
    dct:source <http://inparanoid.sbc.su.se> ;
    pav:version "7.0" ;
    pav:derivedFrom <http://orthoxml.org/0.3/orthoxml_doc_v0.3.html#example> ;
    void:dataDump <http://mbgd.genome.ad.jp/rdf/archive/orthoxml_example.ttl> ;
    orth:organism :worm , :human .

:worm
    a orth:Organism ;
    rdfs:label "Caenorhabditis elegans" ;
    dct:source <http://www.wormbase.org> ;
    pav:version "Caenorhabditis-elegans_WormBase_WS199_protein-all.fa" ;
    orth:taxon taxid:6239 .

:human
    a orth:Organism ;
    rdfs:label "Homo sapiens" ;
    dct:source <http://www.ensembl.org> ;
    pav:version "Homo_sapiens.NCBI36.52.pep.all.fa" ;
    orth:taxon taxid:9606 .

:group1
    a orth:OrthologGroup ;
    dct:identifier "1";
    orth:inDataset :dataset ;
    :bit "5093"^^xsd:integer ;
    :foo "bar" ;
    orth:member :member1 , :member2 .

:group3
    a orth:OrthologGroup ;
    dct:identifier "3";
    orth:inDataset :dataset ;
    orth:member :member5 , :member6 , :member7 .

:member1
    dct:identifier "1";
    :inparalog "1"^^xsd:integer ;
    :bootstrap "1.00"^^xsd:decimal ;
    orth:organism :worm ;
    orth:gene wgene:WBGene00000962 ;
    orth:protein wprot:CE23997 .

:member2
    dct:identifier "2";
    :inparalog "1"^^xsd:integer ;
    :bootstrap "1.00"^^xsd:decimal ;
    orth:organism :worm ;
    orth:gene hgene:ENSG00000197102 ;
    orth:protein hprot:ENSP00000348965 .

:member5
    dct:identifier "5";
    :inparalog "1"^^xsd:integer ;
    :bootstrap "1.00"^^xsd:decimal ;
    orth:organism :worm ;
    orth:gene wgene:WBGene00006801 ;
    orth:protein wprot:CE43332 .

:member6
    dct:identifier "6";
    :inparalog "1"^^xsd:integer ;
    :bootstrap "1.00"^^xsd:decimal ;
    orth:organism :human ;
    orth:gene hgene:ENSG00000198626 ;
    orth:protein hprot:ENSP00000355533 .

:member7
    dct:identifier "7";
    :bootstrap "0.4781"^^xsd:decimal ;
    orth:organism :human ;
    orth:protein hprot:ENSP00000373884 .

:bit
    a owl:DatatypeProperty ;
    rdfs:subPropertyOf orth:groupScore ;
    dct:description "BLAST score in bits of seed orthologs" .

:inparalog
    a owl:DatatypeProperty ;
    rdfs:subPropertyOf orth:memberScore ;
    dct:description "Distance between edge seed ortholog" .

:bootstrap
    a owl:DatatypeProperty ;
    rdfs:subPropertyOf orth:memberScore ;
    dct:description "Reliability of seed orthologs" .

:foo
    a owl:DatatypeProperty ;
    rdfs:subPropertyOf orth:groupValue .
```
