# Supplementary figures and images for: Construction of an Ortholog Database Using the Semantic Web Technology for Integrative Analysis of Genomic Data
Source: PLoS One. 2015 Apr 13;10(4):e0122802. doi: 10.1371/journal.pone.0122802 (PMC4395280; doi:10.1371/journal.pone.0122802)

owl:Class

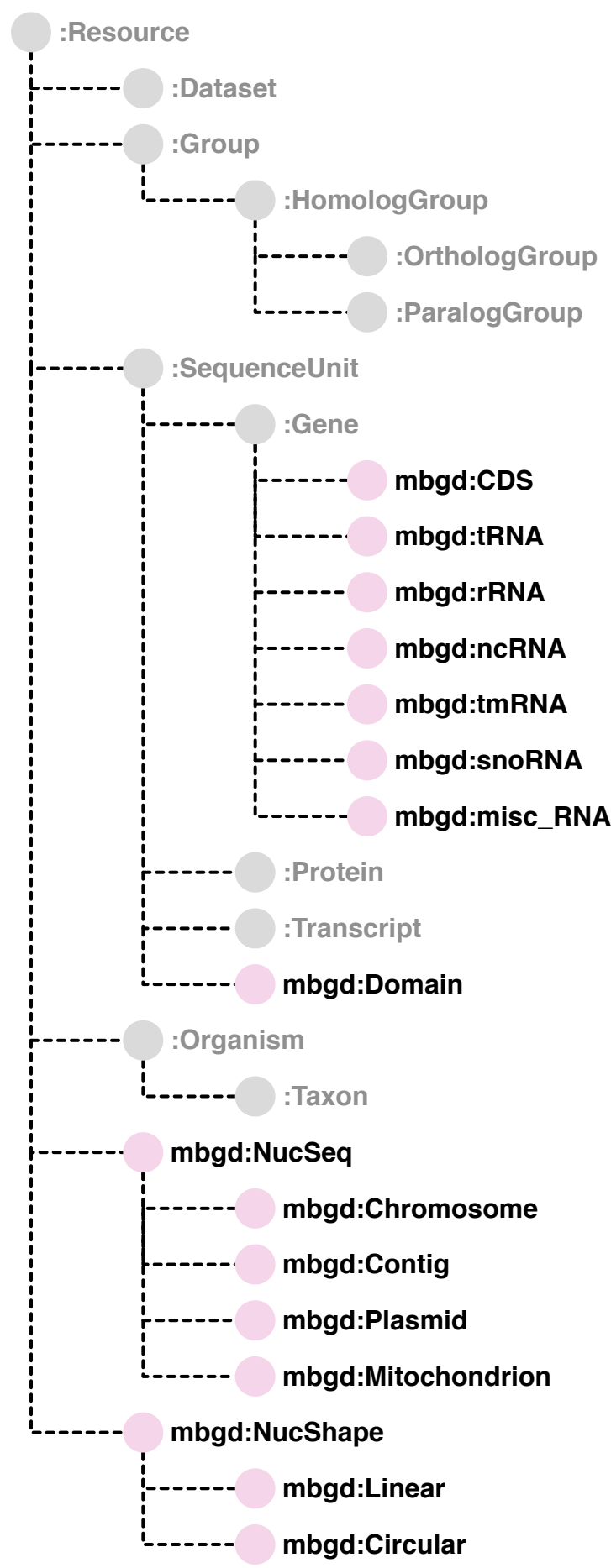

owl:ObjectProperty

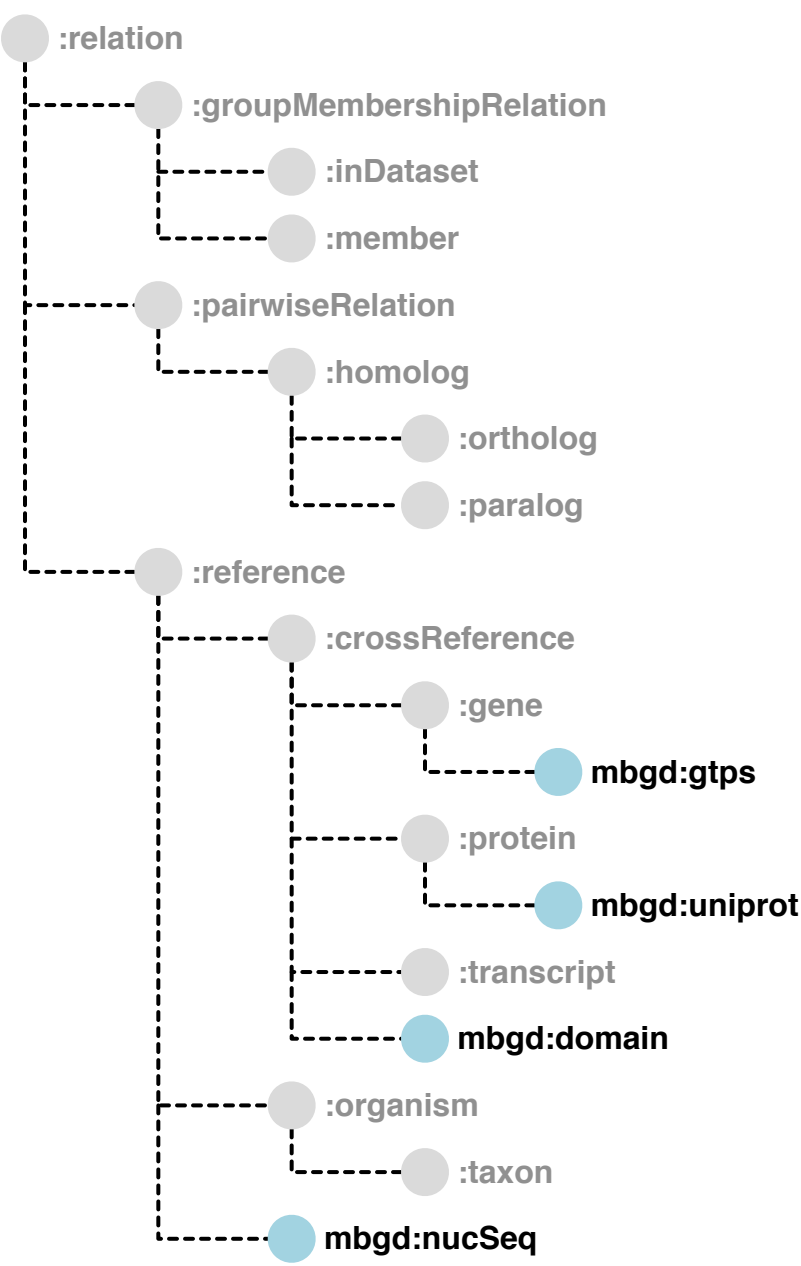

owl:DatatypeProperty

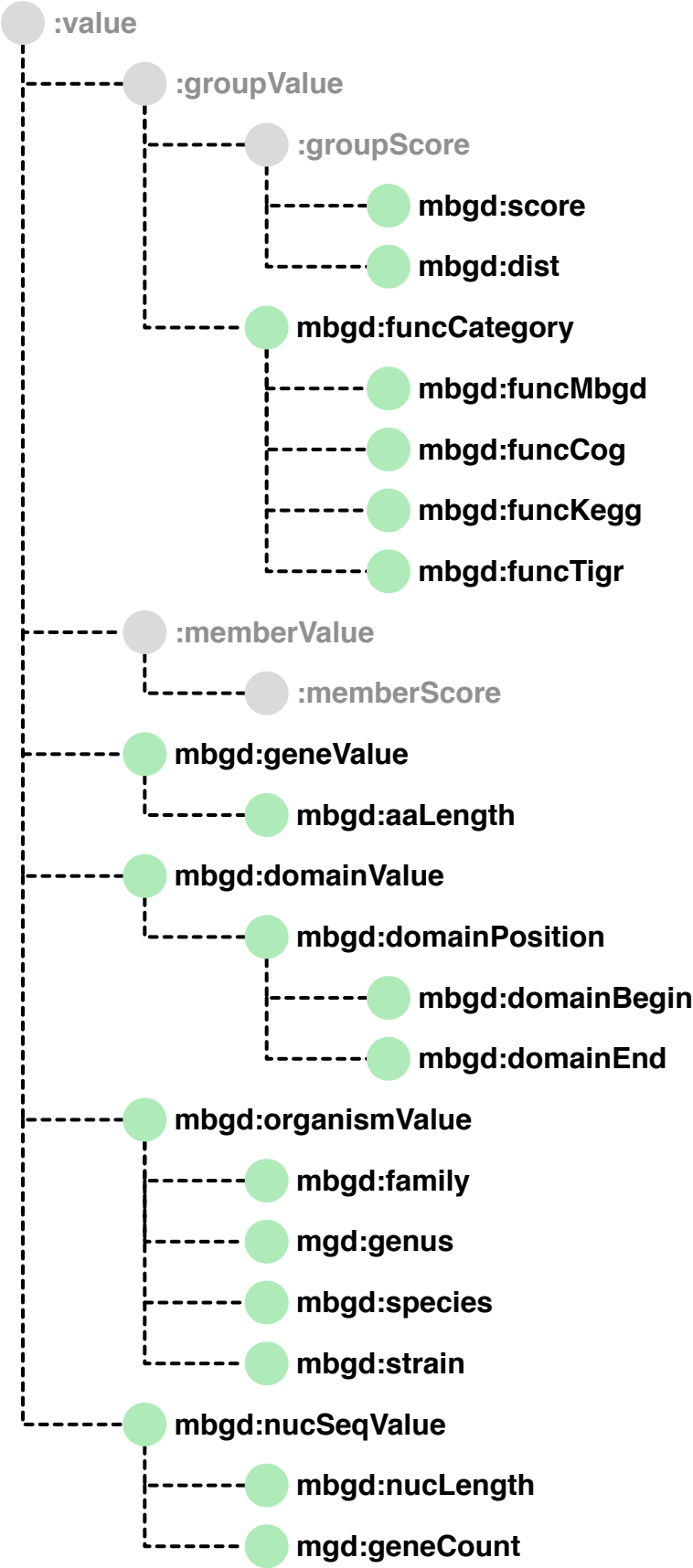

Supplement: S2 Fig — MBGD-O includes 16 classes (owl:Class) and 25 properties (4 of owl:ObjectProperty and 21 of owl:DatatypeProperty). Terms of OrthO are shown in gray. (PDF) [file pone.0122802.s002.pdf]

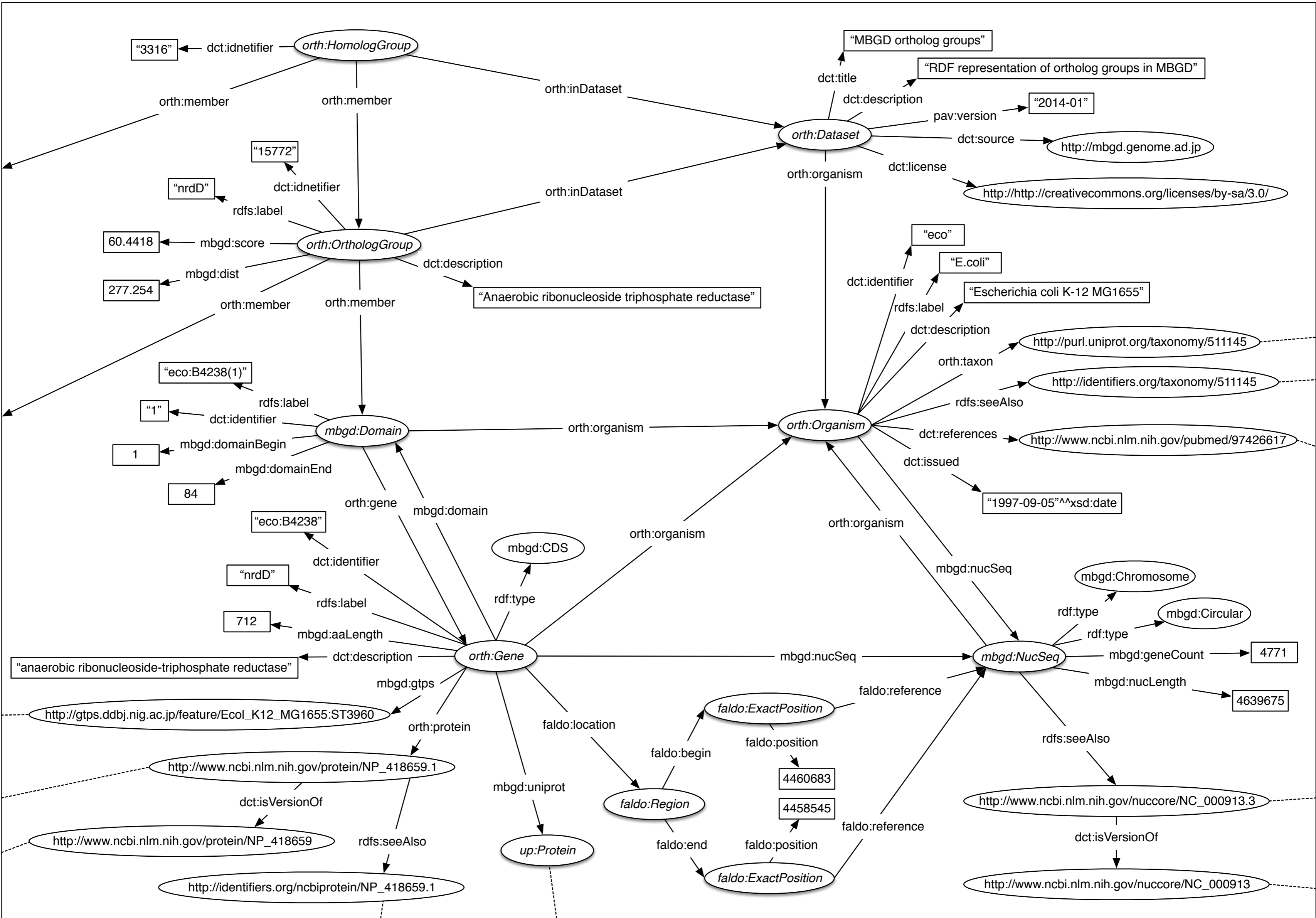

Supplement: S3 Fig — The elliptical nodes represent resources. Specifically, the shaded elliptical nodes where classes are shown in italics represent instances of the classes. In the unshaded elliptical nodes, the URIs of the resources are directly shown. The rectangular nodes represent literals. The directed edges represent properties. The dotted lines represent possible links to other resources. (PDF) [file pone.0122802.s003.pdf]
