# Supplementary material for: Construction of an Ortholog Database Using the Semantic Web Technology for Integrative Analysis of Genomic Data
Source: PLoS One. 2015 Apr 13;10(4):e0122802. doi: 10.1371/journal.pone.0122802 (PMC4395280; doi:10.1371/journal.pone.0122802)
Supplement: S1 Table — (PDF) [file pone.0122802.s004.pdf]

		OrthoXML	OrthO			OGO		
		**<element attribute="">**	**subject**	**predicate**	**object**	**subject**	**predicate**	**object**
**Metadata**	**Data type**	<orthoXML version="*decimal*">	<*datasetURI*>	rdf:type	orth:Dataset			
	**Data source**	<orthoXML origin="*string*">	<*datasetURI*><br><*datasetURI*>	dct:source<br>dc:source	<*dataSourceURI*> *or*<br>*"literal"*	*not included*		
		<orthoXML originVersion="*token*">	<*datasetURI*>	pav:version	*"literal"*			
	**Description of the data**	<orthoXML><notes>	<*datasetURI*>	dct:title	*"literal"*			
			<*datasetURI*>	dct:description	*"literal"*			
**Organism data and cross-references**	**Organism name and ID**	<orthoXML><species name="*literal*">	<*datasetURI*>	orth:organism	<*organismURI*>	<*taxonomyURI*>	rdfs:label	*"literal"*
			<*organismURI*>	rdf:type	orth:Organism			
			<*organismURI*>	rdfs:label	*"literal"*			
		<orthoXML><species NCBITaxID="*integer*">	<*organismURI*>	orth:taxon	<*taxonomyURI*>			
	**Organism data**	<orthoXML><species><database name="*string*">	<*organismURI*><br><*organismURI*>	dct:source<br>dc:source	<*dataSourceURI*> *or*<br>*"literal"*	<*resourceURI*>	rdf:type	ogo:Resource
		<orthoXML><species><database version="*literal*">	<*organismURI*>	pav:version	*"literal"*			
	**Gene / Sequence unit**	<orthoXML><species><database><genes><gene id="*integer*">	<*sequenceUnitURI*>	orth:organism	<*organismURI*>	<*geneURI*>	ogo:fromSpecies	<*taxonomyURI*>
						<*geneURI*>	ogo:hasResource	<*resourceURI*>
	**Gene ID**	<orthoXML><species><database geneLink="*URI*">	<*sequenceUnitURI*>	orth:gene	<*geneURI*>			
		<orthoXML><species><database><genes><gene geneId="*string*">	<*geneURI*>	dct:identifier	*"literal"*	<*geneURI*>	ogo:Identifier	"*literal*"
			<*geneURI*>	rdf:type	orth:Gene	<*geneURI*>	rdf:type	ogo:Gene
	**Protein ID**	<orthoXML><species><database protLink="*URI*">	<*sequenceUnitURI*>	orth:protein	<*proteinURI*>	<*geneURI*>	ogo:isTranslatedTo	<*proteinURI*>
		<orthoXML><species><database><genes><gene proteinId="*string*">	<*proteinURI*>	dct:identifier	*"literal"*	<*proteinURI*>	ogo:Identifier	*"literal"*
			<*proteinURI*>	rdf:type	orth:Protein	<*proteinURI*>	rdf:type	ogo:Protein
	**Transcript ID**	<orthoXML><species><database transcriptLink="*URI*">	<*sequenceUnitURI*>	orth:transcript	<*transcriptURI*>	*not included*		
		<orthoXML><species><database><genes><gene transcriptId="*string*">	<*transcriptURI*>	dct:identifier	*"literal"*			
			<*transcriptURI*>	rdf:type	orth:Transcript			
**Grouping information**	**Score definition**	<orthoXML><scores><scoreDef id="*NCName*">	<*scoreNameURI*>	rdf:type	owl:DatatypeProperty	*not included*		
			<*scoreNameURI*>	rdfs:subPropertyOf	orth:groupScore *or*<br>orth:memberScore			
		<orthoXML><scores><scoreDef desc="*literal*">	<*scoreNameURI*>	rdfs:label	*"literal"*			
			<*scoreNameURI*>	dct:description	*"literal"*			
	**Ortholog group**	<orthoXML><groups><orthologGroup id="*string*">	<*orthologGoupURI*>	rdf:type	orth:OrthologGroup	<*clusterURI*>	rdf:type	ogo:Cluster
			<*orthologGoupURI*>	orth:inDataset	<*datasetURI*>			
			<*orthologGoupURI*>	dct:identifier	*"literal"*			
	**Group score**	<orthoXML><groups><orthologGroup><score id="*NCName*">	<*orthologGoupURI*>	<*scoreNameURI*>	*"literal"*	*not included*		
		<orthoXML><groups><orthologGroup><score value="*decimal*">						
	**Member**	<orthoXML><groups><orthologGroup><geneRef id="*integer*">	<*orthologGoupURI*>	orth:member	<*sequenceUnitURI*>	<*clusterURI*>	ogo:hasOrthologous	<*geneURI*>
	**Member score**	<orthoXML><groups><orthologGroup><geneRef><score id="*NCName*">	<*sequenceUnitURI*>	<*scoreNameURI*>	*"literal"*	*not included*		
		<orthoXML><groups><orthologGroup><geneRef><score value="*decimal*">						
**Other metadata**	**License**	*not included*	<*datasetURI*>	dct:license	<*licenseURI*>	*not included*		
	**Creator**		<*datasetURI*>	dct:creator	<*creatorURI*>			
	**Publisher**		<*datasetURI*>	dct:publisher	<*publisherURI*>			
	**Date and time of creation and publication**		<*datasetURI*>	dct:created *and/or*<br>dct:issued	*"dateTime"*			
